# The Role of Intravenous Immunoglobulin Preparations in the Treatment of Systemic Sclerosis

**DOI:** 10.1155/2011/829751

**Published:** 2011-11-02

**Authors:** Marta Baleva, Krasimir Nikolov

**Affiliations:** ^1^Department of Clinical Immunology, University Hospital Alexandrovska, 1 G. Sofiiski Street, 1431 Sofia, Bulgaria; ^2^Department of Dermatovenereology, Varna Medical University, 55 M. Drinov Street, 9000 Varna, Bulgaria

## Abstract

Scleroderma is progressive autoimmune disease associated with severe disability. The major underlying pathological process in scleroderma is progressive development of fibrous tissue and obliteration of the microvasculature. Currently, there are no medical products for the treatment of scleroderma that provide both sufficient immunosuppression and low-risk side safety profile with negligible side effects. There are a large number of experimental data showing that intravenous immunoglobulin (IVIG) has multiple clinical and morphological effects. On the other hand, some authors report good effect of intravenous immune globulins in patients with scleroderma. The less frequent side effects of IVIG in doses below or equal to 2 g/kg/month divided in 5 consecutive days make IVIG a promising treatment of choice in scleroderma.

In 1981, the Swiss physician Paul Imbach prescribed IVIG to a child with immune deficiency combined with idiopathic thrombocytopenic purpura (ITP). He was surprised that, besides the increase in serum IgG levels, a dramatic improvement in the platelet count was observed. By the middle 1980s, such increase in platelet count in autoimmune thrombocytopenia after the administration of IVIG had been observed by many authors and IVIG were included in the standard treatment for ITP. Moreover, the indications for IVIG were broadened, and these preparations were administered in a wide range of diseases.

**Rationale for the Use of IVIG**

1. IVIG has immunomodulatory action.
2. IVIG contains idiotypes that neutralize different autoantibodies.
3. IVIG blocks the Fc receptors on the surface of B-cells and macrophages.
4. IVIG inhibits inflammatory mediators, such as cytokines, chemokines, and metalloproteinases.
5. IVIG neutralizes toxins.
6. IVIG reduces immune complexes.
7. Substitutive treatment in immune deficiencies.
8. Alternative treatment, for example, in cases where all other immunosuppressive therapeutic modalities have shown to be inefficient (*ultima ratio*).

Food and Drug Administration (FDA) has licensed the administration of IVIG for the following six therapeutic indications:

1. treatment of primary immune deficiencies;
2. prevention of infectious complications in patients with chronic B-cell lymphatic leukemia and hypogammaglobulinemia;
3. prevention of coronary aneurism in Kawasaki's disease;
4. prevention of infections and graft-versus-host disease after bone marrow transplantation;
5. minimization of the risk for severe bacterial infections in children with HIV-infection;
6. increasing of the platelet count and prevention of hemorrhages in patients with ITP.

In dermatology, IVIG is used mainly in patients with dermatomyositis and polymyositis [1, 2] and with autoimmune bullous dermatoses [3–5]. Recently, beneficial effect in small series of patients with different forms of scleroderma has been reported by some authors.

Scleroderma is progressive autoimmune disease associated with severe disability. It is manifested as diffuse cutaneous (dcSSc), limited cutaneous (lcSSc), or disease with multisystemic and multiorgan involvement (SSc). The major underlying pathological process in scleroderma is progressive development of fibrous tissue and obliteration of the microvasculature. Currently, there is no specific medicinal treatment of scleroderma that could provide both sufficient immunosuppression and low-risk side safety profile with negligible side effects. Several cytotoxic and immunomodulatory agents are used for the treatment of scleroderma, including cyclophosphamide, mycophenolate mofetil, cyclosporine, and, more recently, selective inhibitors of the T-(Sirolimus, Alefacept) and B-cells (Rituximab), antifibrotic agents (Imatinib), and hematopoietic stem cell transplantation. 

In 1990, Bodemer et al. report the beneficial effect of IVIG 2 g/kg/month combined with prednisone for 10 months in patients with SSc-dermatomyositis overlap [6]. They point that the skin lesions are only on the face and the improvement after IVIG concerns only the myositis (Table 1). In 1998, Wollina et al. reported the beneficial effect of IVIG on the satellite infection in a patient with disabling morphea [7]. In 2000, Levy et al. report the beneficial effects of IVIG 2 g/kg/month in three patients with SSc [8]. All of them had past history for progressive and rapidly deteriorating skin symptoms not responsible to previous therapy with colchicine and one of them—colchicne and D-penicillamine. Two of the patients had been treated for 6 months without side effects, and, in the third patient, the treatment was ceased after month 3 due to renal damage and subsequent sepsis. In all three patients, regression in the skin score and disease stabilization were observed, but the levels of PM-Scl antibodies measured by indirect immunofluorescence test showed no changes during treatment (Table 1). Discussing these data, the authors point that “three patients are insufficient to evaluate a novel therapy.” They assume that future studies are needed to show the effect of IVIG on scleroderma.

In 2004, a research group from the Sheba Medical Center and the University of Florence [9] reported 15 patients with SSc (5 with lcSSc and 10 with dcSSc) treated with IVIG 2 g/kg/month (Table 1). Eleven patients had been treated for 6 months, three for 4 months, and one patient for 3 months. In all patients, the skin involvement was evaluated using the modified Rodnan skin score (mRSS) at baseline and after the completion of treatment. Eight patients completed the Health Assessment Questionnaire (HAQ). The mean mRSS after therapy with IVIG was significantly decreased: shorter disease duration was associated with milder degree of improvement (21% in mRSS), longer disease duration (≥2 years)—44% in mRSS. Moreover, the HAQ score revealed that the patients report marked improvement. The authors assume that suppressing the action of profibrotic cytokines (IL-4, TGF beta) IVIG may improve the disease and quality of life of patients with SSc. 

Several years later in a pilot study, the same authors reported marked improvement in articular involvement in seven Caucasian patients with severe refractive arthropathy in SSc (five with localized and 2 with diffuse systemic sclerosis) treated with IVIG 2 g/kg/month during 4 days/month for six consecutive courses [10]. The authors evaluated the effect of the treatment upon the following symptoms: joint tenderness and swelling and articular deformities. At baseline and after six-month treatment, the patients underwent the following tests: Ritchie Index (RI) evaluation of articular involvement; Dreiser Algo-Functional Index (IAFD) evaluation of hand joint function; pain visual analogue scale (VAS) to measure joint pain; HAQ to evaluate the limitations in everyday living and physical disability; mRSS for evaluation of skin involvement. After six-month therapy with IVIG in 6/7 patients VAS, IAFD and mRSS decreased significantly and HAQ score showed improvement in general functionality (Table 1). According to the authors, the therapy with IVIG may be useful for SSc patients with severe joint involvement and those who are refractory to other treatment.

 In 2005, Asano et al. reported similar results in a 60-years-old female Japanese patient with diffuse scleroderma treated with IVIG 400 mg/kg for 5 consecutive days [11]. The patient's condition improved dramatically with significant decrease in mRSS and marked improvement on the histological examination (Table 1). 

In 2009, Szekanecz et al. reported a case of diffuse SSc without response to conventional therapy. After 12 months of combined repeated treatment with IVIG and plasmapheresis, they administered a good clinical effect [12]. 

There is a large number of experimental data showing that IVIG has multiple clinical and morphological effects. For instance, the administration of IVIG 2 g/kg in tight-skin mouse leads to significant decreases in collagen depositions and reduction of type I collagen gene expression [13].

It has been speculated that the overproduction of fibrotic tissue in SSc is due to the increased expression of TGF*β*1 and IL-4 [14, 15]. The experimental studies of Blank et al. in tight-skin mice (animal model of SSc) revealed that the administration of IVIG in total dose 2 g/kg for four weeks lead to marked decrease in collagen deposition and reduction in type I collagen gene expression [13]. The authors observed parallel inhibition of TGF*β*1 and IL-4 secretion by splenocytes with no changes in IFN*γ* levels. The studies of Asano et al. [11] revealed that the fibroblasts of scleroderma patients contain more type I procollagen, TGF*β* and *α*-smooth muscle actin, and less matrix metalloproteinase 1. After the administration of IVIG the initial abnormalities normalize. Amemiya et al. [16] speculate that in inflammatory myopathies IVIG can bind directly to TGF*β*. On the other hand, IVIG downregulate T cells with subsequent decrease in fibroblast TGF*β* production. Other possible explanations of the beneficial effect of IVIG in reducing of fibrosis include inhibition of complement cascade, presence of anti-Fas antibodies in IVIG preparations that inhibit fibrogenesis by blocking the activity of Fas, and presence of antifibroblast antibodies that inhibit fibrogenesis [17]. These experimental data confirm the hypothesis that the treatment with IVIG in patients with different forms of scleroderma could change the fibroblast phenotype in this disease. 

There are not so many studies about the effect of IVIG in SSc patients. We found only several publications, of which mainly case reports discussing the beneficial effect of this treatment in SSc. On the other hand, the less frequent side effects of IVIG in doses below or equal to 2 g/kg/month divided in 5 consecutive days make IVIG a promising treatment of choice in patients with scleroderma refractory to other therapy. The current use of IVIG in dermatology is at a rise, and this demands for new controlled clinical studies in different autoimmune skin diseases including SSc. 

## Figures and Tables

**Table 1:** IVIG in patients with SSc.

Author/year	Number of patients	Skin lesions	Results	Remarks
Bodemer et al. 1990 [6]	1	Only in the face	Myositis improvement	SSc-dermatomyositis overlap
Levy et al. 2000 [8]	3	dcSSc	Regression of skin score, disease stabilization	No changes in PM-Scl antibodies
Levy et al. 2004 [9]	15	5 patients-lcSSc,10 patients-dcSSc	Decreased mRSS, changes in HAQ score	Very high skin score in patients with lcSSc
Nacci et al. 2007 [10]	7	5 patients-limited SSc, 2 patients diffuse SSc	Decreased VAS, IAFD, mRSS after 6-month treatment	Effects on joints and skin
Asano et al. 2005 [11]	1	Fingers, hands, forearms, upper arms, face, chest, abdomen, lower legs, dorsum of the feet	Decreased mRSS	
Szekanecz et al. 2009 [12]	1	Disuse SSc, refractory to treatment	Good clinical effect	Combination of IVIG and plasmapheresis
